# Augmented Total Elbow Arthroplasty with Femoral Strut Allograft for Revision of Prosthetic Joint Infection with Distal Humerus Bone Loss and Incomplete Union of Periprosthetic Humeral Shaft Fracture

**DOI:** 10.1155/2021/6661870

**Published:** 2021-04-21

**Authors:** William R. Monahan, Elizabeth Alaimo, Jared M. Mahylis

**Affiliations:** ^1^Department of Orthopaedic Surgery, Midwestern University/Franciscan Health-Olympia Fields, 20202 S. Crawford Ave, Olympia Fields, IL 60461, USA; ^2^Department of Family Medicine, Midwestern University/Franciscan Health-Olympia Fields, 3700 West 203rd Street, Olympia Fields, IL 60461, USA

## Abstract

Total elbow arthroplasty (TEA) prosthetic joint infection (PJI) in the setting of distal humerus bone loss poses a challenge for restoration of function. This can be complicated by a periprosthetic humeral fracture. Revision surgery in the setting of these pathologies possesses a significant challenge, especially when two or, in this case, all three problems are treated simultaneously. We present the clinical course, operative findings, and definitive treatment with the use of an augmented total elbow arthroplasty and femoral strut allograft reinforcement in detail. A review of the literature regarding the identification and management of infected TEA with augmented prosthesis and bone allograft augmentation of humerus fractures will be outlined in this case report.

## 1. Introduction

The prevalence of total elbow arthroplasties (TEA) has increased over the last several years [1, 2]. While TEA was classically used to manage end-stage rheumatoid arthritis (RA) [3], indications have expanded over the last few decades to include treatment of posttraumatic arthritis, irreparable acute fractures, and less commonly oncologic reconstruction [3]. As indications have evolved, so too has implant design to address these needs [4, 5]. Despite these advances, there remains a high incidence of complications associated with TEA including periprosthetic joint infection (PJI), instability, and component failure, as well as periprosthetic fracture [6]. PJI remains one of the most devastating complications with an incidence of 1-12% in primary surgeries and recurrence ranging from 28% to 50% [7, 8]. The question of the best treatment course in PJI treatment remains debated [9]. Frequently, revision surgery is performed, either in a single- or two-stage procedure [7, 8, 10–12]. Literature has shown positive outcomes with revision arthroplasty [11]. Adding to revision challenges, a periprosthetic fracture of TEA remains a significant complication with incidence at nearly 5% [13], and occurrence during revision TEA can be substantial [14]. Strut allograft augmentation of a periprosthetic fracture has shown good results in association with TEA [14]. Furthermore, bone loss is also a challenging hurdle for revision. Restoration of soft tissue tensioning is necessary to reestablish both stability and function. Traditionally, an allograft prosthetic composite has served as a reasonable and durable option [1], while more recently use of augmented megaprosthesis has provided further options to restore bone loss at the time of revision surgery [5]. Herein, we describe a case of an infected total elbow with distal humerus bone loss exacerbated by a periprosthetic fracture at the time of first-stage revision surgery resulting in incomplete humeral union. The clinical course including preoperative workup and staged and definitive treatment will be presented. A review of the available literature regarding identification and management of infected TEA with augmented/megaprosthesis and bone allograft augmentation of humerus fractures is presented.

## 2. Case Presentation

**Figure 1:**
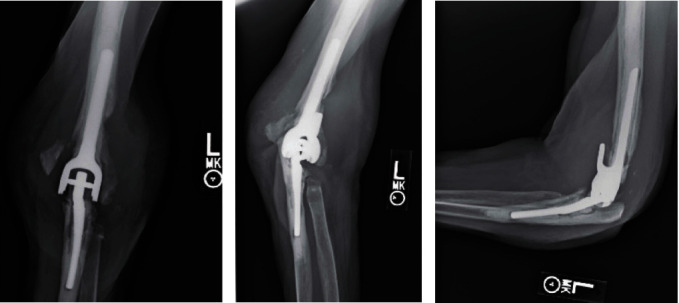
(a–c) Preoperative X-ray imaging of the operative left elbow showing prosthetic loosening and periprosthetic fracture. (a) AP imaging of the left elbow with new periprosthetic fracture of the medial epicondyle and significant soft tissue swelling. (b) Oblique imaging of the left elbow again demonstrating a periprosthetic fracture of the medial epicondyle and lucency around the lateral aspect of the humeral implant. (c) Lateral X-ray of the left elbow demonstrating both ulnar and humeral component lucency concerning for infection.

The patient is a left-hand-dominant 75-year-old female with history of prior cerebral vascular accident without residual left upper extremity deficits, Crohn's disease, hypertension, and mitral valve prolapse who underwent TEA in November 2018 for the treatment of end-stage arthritis. Total elbow arthroplasty was performed at an outside facility by another surgeon. Three months following primary surgery, the patient reported persistent swelling, erythema, and pain of the operative elbow. Per patient report, multiple aspirations were performed, but she denies being given a diagnosis for or undergoing treatment for PJI. She initially presented to us in July 2019 for evaluation with complaint of unrelenting elbow pain, erythema, and swelling. Physical exam revealed diffuse induration and erythema over the posterolateral left elbow, without draining sinus, limited ROM of the left elbow of 30-90°, and no neurovascular deficits. Radiographic imaging demonstrated signs of loosening of both humeral and ulnar components, ulnar osteolysis concerning for infectious etiology, and a new periprosthetic fracture of the medial epicondyle (Figure 1). Infectious workup was completed with initial erythrocyte sedimentation rate (ESR) elevated to 47 mm/h (normal 0-30 mm/h), but normal C-reactive protein (CRP) at 0.3 mg/dl (normal 0.0-0.9 mg/dl) and no leukocytosis evident on Complete Blood Count (CBC) (white blood count 5.1 × 10^3^/*μ*l). Due to continued concern for infection, the patient was indicated for left elbow irrigation and debridement (I&D), removal of implants, possible revision elbow arthroplasty, and possible placement of an antibiotic spacer. Antibiotics were held prior to surgery until cultures and intraoperative frozen sections had been obtained.

**Figure 2:**
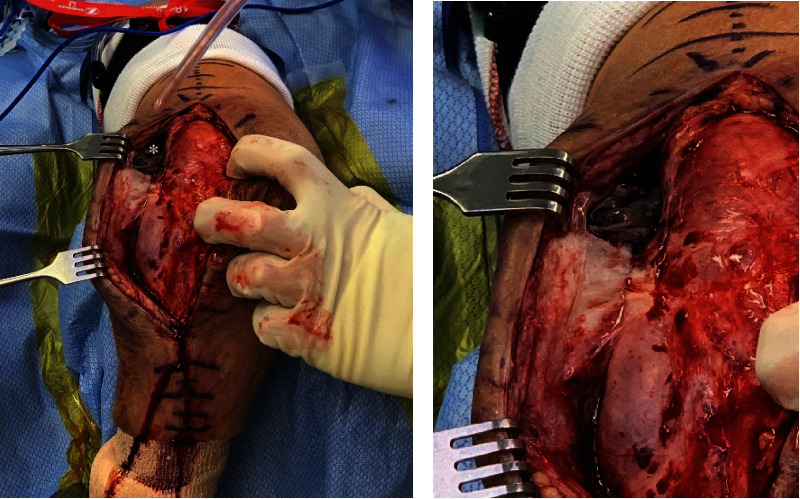
(a, b) Intraoperative images showing active infection at the time of the first stage of revision surgery. (a) Posterior aspect of the left arm and elbow with abscess with a lateral paratricipital region and direct communication to a joint (white ∗) and (b) close-up of walled off abscess of the left elbow.

Initial revision surgery was performed in August 2019. The patient was placed in a lateral decubitus position. A triceps tongue approach described by Marinello et al. was performed through the previous posterior surgical incision [15]. Dissection into subcutaneous tissue exposed a purulent fluid collection with signs of communication to the underlying joint (Figure 2). Capsulotomy was performed confirming communication between the joint and abscess as well as notable inflamed and infected synovium and early metallosis. A total of 4 tissue culture specimens and one fluid culture were sterilely obtained. Tissue samples were also sent for a frozen section which yielded >10 WBC per high-power field. At this time, a two-stage arthroplasty with an antibiotic spacer was performed. The ulnar component was found to be grossly loose as it could be removed by hand during extraction. The humeral component was found to be moderately fixed with some implant motion at the cement interface. The humeral implant could not be removed by hand. The humeral component and cement mantel were removed with a combination of high-speed burr and cement extraction tools. However, during removal of the cement mantel, the periprosthetic fracture of the medial column propagated causing a spiral fracture of the humeral shaft. The remaining intact humeral canal and proximal ulna were again irrigated and debrided. A hand-molded antibiotic spacer was prepared, consisting of two bags of gentamicin-impregnated antibiotic cement, mixed with a total of 4 grams of vancomycin. As the surgical bed had gross infection signs, staged treatment of the infected TEA with an antibiotic spacer and closed treatment of the humerus fracture with a Sarmiento brace and bone stimulator was chosen rather than immediate fixation [16, 17].

Postoperatively, the patient was managed by the infectious disease service and was placed on IV vancomycin and oral levofloxacin daily for 6 weeks. All tissue and the single fluid culture (5 of 5 cultures) were positive for *Staphylococcus epidermidis* (*Staph epi*).

**Figure 3:**
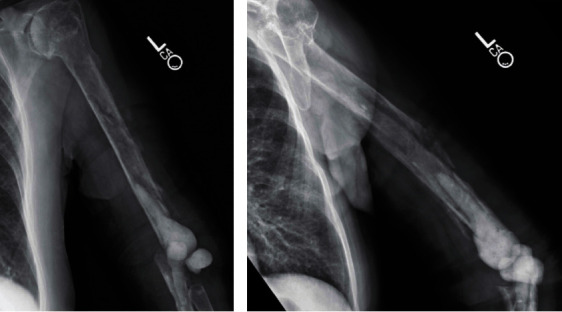
(a, b) Five-month postoperative images following first-stage revision surgery. (a) AP humeral X-rays showing delayed healing of the humeral shaft fracture and antibiotic spacer of the humerus, ulna, and elbow joint. (b) Lateral humerus X-ray showing incomplete healing of the humeral shaft fracture.

**Figure 4:**
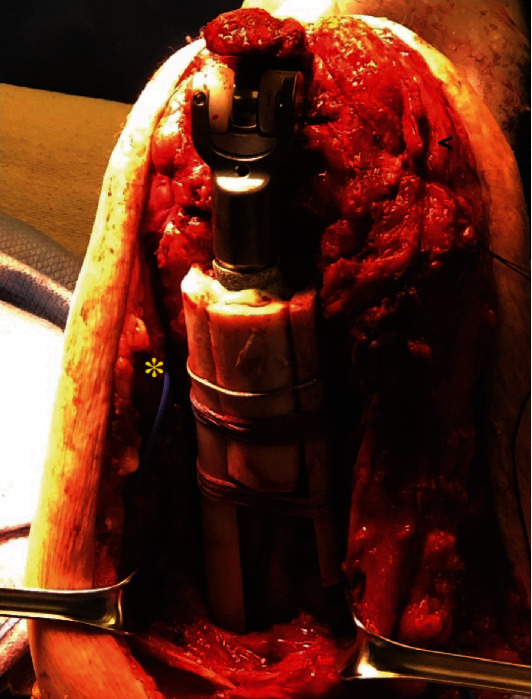
Intraoperative images showing a “sandwich” technique of the femoral strut allograft and total elbow arthroplasty with 50 mm distal augment. Triceps tendon (black <) released off the ulna in a continuous sleeve with anconeus muscle. Ulnar nerve (yellow ∗) marked with a vessel loop to ensure protection throughout the procedure.

At 5-month follow-up, the patient had minimal left elbow pain but notable functional limitations. Computed tomography (CT) imaging (not available) and radiographs revealed a left humeral shaft fracture with incomplete union (Figure 3). Infectious workup with elbow synovial fluid *α*-defensin, ESR (18 mm/h), and CRP (0.1 mg/dl) was within normal limits. Further staged treatment with surgical fixation of the fracture nonunion prior to TEA was offered to the patient; however, as this was her dominant extremity, she opted for alternative surgical options. Earlier surgery was delayed to allow for increased healing of the periprosthetic humerus shaft fracture. The patient returned to the OR for repeat I&D, open surgical fracture fixation with femoral strut allograft augmentation of the humeral shaft nonunion, and total elbow arthroplasty. The previous posterior elbow incision was again used; however, a triceps-off approach described by Morrey [18] was utilized to allow for mobilization of soft tissue and effective proximal neurolysis of the radial and ulnar nerves. The antibiotic spacers were removed. A total of 4 tissue cultures and one fluid culture were obtained. The humerus was noted to have a stable fibrous tissue and incomplete union, but the humeral canal was patent. The femoral allograft struts were contoured with a burr, and the native humerus cortical surface was gently prepared with a burr. The allograft struts were placed along medial (146 mm), lateral (112 mm), and posterior aspects (61 mm) of the humerus similar to previously described techniques [14, 19]. Allograft was used to augment the incomplete union of the humerus. FiberWire cerclage cables (Arthrex, Naples, FL, USA) were used to secure the allografts near the neurovascular structures as well as a 1.7 mm stainless-steel cerclage cable (DePuy Synthes, Warsaw, IN, USA) which was utilized for a radiographic marker to monitor for early loosening. A cement restrictor was placed within the native humeral canal. PALACOS gentamicin-impregnated cement (Heraeus Medical, Yardley, PA, USA) was placed in the humeral canal and an SRS augmented total elbow arthroplasty (Zimmer-Biomet, Warsaw, IN, USA) with a 6 mm × 75 mm humeral stem, and a size of 50 mm augment was cemented in place. A 75 mm × 4 mm Nexel ulnar component (Zimmer-Biomet, Warsaw, IN, USA) was cemented in place. The elbow was reduced and components were linked (Figure 4). The elbow motion was 0-130 degrees of extension-flexion and 80-80 degrees of pronation-supination without evidence of instability or implant stress. The triceps was reattached to the proximal ulna using FiberWire nonabsorbable suture (Arthrex, Naples, FL, USA) as previously described [18]. The patient was placed on prophylactic antibiotics with doxycycline for 2 weeks until cultures resulted. A single intraoperative culture was positive for *Cutibacterium acnes* at 12 days and *Staphylococcus capitis* at 14 days, both susceptible to doxycycline. Antibiotics were continued for an additional month, for a total of 6 weeks.

**Figure 5:**
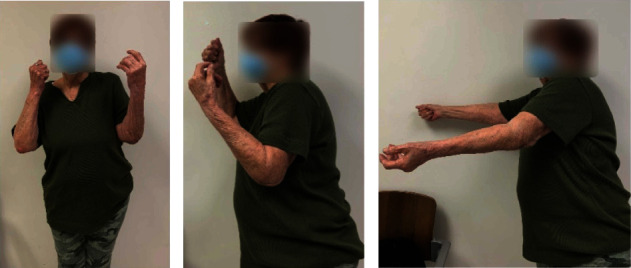
(a–c) 9-month postoperative clinic photos showing functional motion (10-130 degrees). (a, b) Symmetric elbow flexion with 130 degrees of the surgical elbow and 140 of the contralateral elbow. (c) Symmetric elbow extension with the surgical (left) elbow demonstrating 10 degrees short of full extension compared to the full extension of the contralateral elbow.

**Figure 6:**
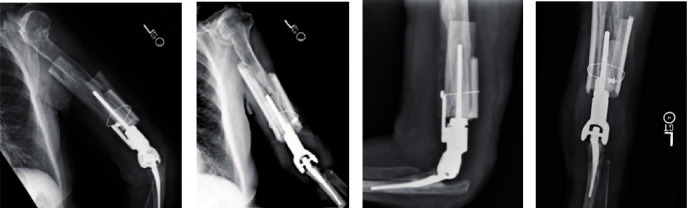
(a–d) 12-month postoperative X-ray images: (a) lateral humerus X-ray; (b) AP humeral X-ray; (c) lateral elbow X-ray; (d) AP elbow X-ray.

Postoperatively, the patient improved significantly and at 12 months after the second-stage revision surgery had no pain in her elbow. The Mayo Elbow Performance Score (MEPS) at nine months was 90 points (range 0-100). Her active arc of motion was excellent: 10°-130° (Figure 5), and X-ray showed stable TEA components without signs of failure or loosening or resorption of the strut allograft (Figure 6). The patient is scheduled for a follow-up at 2 years from her revision surgery.

## 3. Discussion

This case exemplifies the challenges of TEA PJI in conjuncture with periprosthetic fracture and bone loss. Rates of elbow arthroplasty have increased 44% in the United States, particularly with acute trauma or in posttraumatic arthritis [2]. With increasing number of procedures, the risk of complications and resulting revision surgery will increase. Infection in TEA has historically been higher than that in other arthroplasties [12]. As with our case, *Staph epi* remains one of the most common pathogens of deep infection, accounting for nearly 45% of cases [20, 21] with *Staphylococcus aureus* being the other dominant pathogen with 25-47% incidence [7, 8, 11]. Yamaguchi et al. assessed 25 patients with TEA PJI and found only 7 of 14 patients treated with I&D, and retention of implants had successful outcomes and treatment failed in four of four patients with *Staph epi* [21].

Revision surgery with single or two stages has shown moderate to good short-term results [7, 8, 10, 11]. Peach et al. found that patients who underwent a second-stage revision without recurrent infection had a mean Mayo Elbow Performance Score (MEPS) of 81.1 (65 to 95) [7]. However, studies remain limited by small population size and duration of follow-up with one study only noting 68.4% infection-free survival at 3 years [8].

Bone loss and periprosthetic fracture present another challenge at the time of revision. The use of large segment allograft struts and/or allograft prosthetic composite (APC) has shown efficacy for supporting weak bone as in fracture or replacing deficient bone [1, 14]. Sanchez-Sotelo et al. found mean MEPS of 79 points (range, 40 to 100 points) with a mean range of motion from 16° (range, 0° to 30°) of extension to flexion of 131° (range, 110° to 140°) in 10 patients treated with strut allograft for the TEA periprosthetic fracture [14]. Similarly, in the setting of bone loss, Morrey et al. found a mean MEPS of 84 in 25 patients undergoing revision TEA with APC [1].

Megaprosthesis (distal humerus replacement) has more recently become an option for severe bone loss around the elbow. Henrichs et al. in an assessment of 12 patients treated with distal humerus replacement found that patients achieve good functional results, although the complication rate, predominantly loosening rate of the humeral stem, remained high and implant survival at 5 years was 64% [5].

It is difficult to treat periprosthetic TEA fractures in the setting of infection. Based on the Mayo classification, our fracture could be considered type III [12]. While alternative treatment options in the form of surgical fracture fixation with plate osteosynthesis and even long segmental prosthetic replacement remains viable treatment, this ultimately is not what the patient selected after discussion of those options.

Our patient demonstrated three simultaneous issues with PJI, bone loss, and incomplete healing of the periprosthetic fracture. We believe that the staged revision in combination with increased fracture support utilizing strut allograft to reinforce native bone and augmented total elbow allowed for improved strength and implant stability while allowing early return of function. Though staged treatment of the periprosthetic fracture prior to revision elbow arthroplasty could be considered, as this was the patient's dominant extremity, we believe that this technique offered her an expedited recovery with adherence to previously successful techniques [5, 8, 14, 19].

## 4. Conclusion

In summary, this case report discusses a unique complication of PJI, periprosthetic fracture, and bone loss in a non-weight-bearing joint. With a combination of multiple techniques, revision surgery resulted in a good outcome.
